# Supplementary material for: Influence of lower limb isokinetic muscle strength and power on the occurrence of falls in community-dwelling older adults: A longitudinal study
Source: PLoS One. 2024 Apr 4;19(4):e0300818. doi: 10.1371/journal.pone.0300818 (PMC10994367; doi:10.1371/journal.pone.0300818)
Supplement: Attachment — Submitted filename: Response to Reviewers.docx [file pone.0300818.s002.docx]

Mehrnaz Kajbafvala, Ph.D

Academic Editor

PLOS ONE

February 16, 2024

Dear Editor,

Revision of the Manuscript Number: PONE-D-23-25022

Title: **"** **Influence of lower limb isokinetic muscle strength and power on the occurrence of falls in community-dwelling older adults: a longitudinal study".**

Thank you for the opportunity to resubmit our manuscript. The comments from the reviewers were highly insightful and enabled us to improve the quality of our manuscript. We have changed it according to their advice. The responses to the reviewers are attached, as well as the manuscript's marked-up version (highlighted in yellow).

Thank you again for your time and effort in considering this manuscript for publication.

Yours sincerely,

**Journal Requirements:**

When submitting your revision, we need you to address these additional requirements.

**1. Please ensure that your manuscript meets PLOS ONE's style requirements, including those for file naming. The PLOS ONE style templates can be found at**

**https://journals.plos.org/plosone/s/file?id=wjVg/PLOSOne_formatting_sample_main_body.pdf and**

**https://journals.plos.org/plosone/s/file?id=ba62/PLOSOne_formatting_sample_title_authors_affiliations.pdf**

**Answer:** You are very welcome for the review opportunity. We have implemented the requested changes as per the provided instructions.

**2. Note from Emily Chenette, Editor in Chief of PLOS ONE, and Iain Hrynaszkiewicz, Director of Open Research Solutions at PLOS: Did you know that depositing data in a repository is associated with up to a 25% citation advantage (https://doi.org/10.1371/journal.pone.0230416)? If you’ve not already done so, consider depositing your raw data in a repository to ensure your work is read, appreciated and cited by the largest possible audience. You’ll also earn an Accessible Data icon on your published paper if you deposit your data in any participating repository (https://plos.org/open-science/open-data/#accessible-data).**

**Answer:** We appreciate your valuable suggestion; however, at the moment, we are not interested in pursuing the proposal. All data will be made available upon request to the corresponding author. Thank you for your understanding.

**3. Thank you for stating in your Funding Statement:**

**"This study was financed in part by the Postgraduate Department from the University of Brasília (SEI N° 23106.102043/2017-01), the Deanery of research from the State University of Goiás (CCB 01/2018), “Coordenação de Aperfeiçoamento de Pessoal de Nível Superior—Brasil” (CAPES) (Finance Code 001), “Fundação de Apoio a Pesquisa do Distrito Federal” (FAPDF) (grant number 00193-00000866/2023-6, 00193.00000773/2021-72, 00193.00000859/2021-3; 00193.00001222/2021-26), and the National Council for Scientific and Technological Development (CNPq; process numbers 309435/2020-0 and 310269/2021). The funders had no role in study design, data collection and analysis, decision to publish, or preparation of the manuscript."**

**Please provide an amended statement that declares *all* the funding or sources of support (whether external or internal to your organization) received during this study, as detailed online in our guide for authors at http://journals.plos.org/plosone/s/submit-now. Please also include the statement “There was no additional external funding received for this study.” in your updated Funding Statement.**

**Please include your amended Funding Statement within your cover letter. We will change the online submission form on your behalf.**

**Answer**: Thank you for the observations. We have incorporated the requested changes in the cover letter and in the Funding section of the manuscript, page 19, lines 280 to 290.

“This study was partially funded by the Postgraduate Department from the University of Brasília, https://dpg.unb.br/ (SEI N° 23106.102043/2017-01 author CAN), the Deanery of research from the State University of Goiás, https://ueg.br/prp/ (CCB 01/2018 author TCDSH), “Fundação de Apoio a Pesquisa do Distrito Federal” (FAPDF), https://www.fap.df.gov.br/ (grant number 00193-00000866/2023-6 author PAG, 00193.00000773/2021-72 author JLQD, 00193.00000859/2021-3 author JLQD; 00193.00001222/2021-26 author JLQD; 00193-00001261/2021-23 author JLQD), and the National Council for Scientific and Technological Development (CNPq), https://www.gov.br/cnpq/pt-br (process numbers 309435/2020-0 and 310269/2021 author JLQD). The funders had no role in the study design, data collection and analysis, decision to publish, or preparation of the manuscript. There was no additional external funding received for this study.”

**4. Please include captions for your Supporting Information files at the end of your manuscript, and update any in-text citations to match accordingly. Please see our Supporting Information guidelines for more information: http://journals.plos.org/plosone/s/supporting-information.**

[Note: HTML markup is below. Please do not edit.]

**Answer:** We apologize for the oversight. The material sent was incorrectly categorized as supporting information. Now, the revised version we submitted it correctly.

**Reviewers' comments:**

**Reviewer #1**

**Reviewer #1: This study was aimed to evaluate Influence of lower limb isokinetic muscle strength and power on the occurrence of falls in community-dwelling older adults. Authors concluded that hip abductor strength and hip flexor power can be considered protective factors against falls in independent older adults in the community. These findings may help in the development of effective fall-prevention strategies for older adults. Overall, the study is interesting, however there are some clarifications needed.**

**Answer**: Thank you for the feedback and the time spent carefully reading our manuscript.

**Comment#1**

**Abstract, Line 31-33, considering the design of this study and the analyses performed, it seems that the statement of dose-response relationship may not appropriate. Please edit it throughout the manuscript.**

**Answer**: Thank you for the feedback. We have incorporated the requested changes throughout the manuscript, specifically in the abstract section, *page 2, line 31*; introduction section, *page 4, line 79*; discussion section, *page 17, line 238*.

**Comment#2**

**Keywords, the keywords should be reflexive of the study performed. Please edit Keywords according to the MESH.**

**Answer:** Thank you for the feedback, however our keywords reflect the main outcomes of the study and are already in accordance with MeSH. Please find below our keywords according to MESH:

Accidental Falls: https://www.ncbi.nlm.nih.gov/mesh/68000058

Muscle Strength: https://www.ncbi.nlm.nih.gov/mesh/68053580

Aged: https://www.ncbi.nlm.nih.gov/mesh/68000368

**Comment#3**

**Introduction, the authors should provide more explanations for investigating the influence of strength and power of the all eight muscle groups in the lower limbs on falls in older adults.**

**Answer:** Thank you sincerely for your consideration and feedback. We appreciate your observations and have incorporated the requested changes in the introduction section, specifically on *page 4, from lines 78 to 102*. We hope the additional explanations regarding the influence of strength and power of all eight muscle groups in the lower limbs on falls in older adults are clearer.

*“While the association between muscle strength, muscle power, and falls has been demonstrated in previous studies* [1–14]*, the specific influence of each lower limb muscle group's strength and power on the occurrence of falls in older individuals remains unclear. Some studies suggest that ankle muscle strength and power are more closely associated with falls in older adults* [1,2,7–11,15]*. However, other research indicates that knee flexors* [12,13]*, knee extensors* [12–14]*, hip flexors* [6]*, hip extensors* [6,13,14]*, hip abductors* [6,12]*, and hip adductors* [6] *also play crucial roles in this outcome.*

*Notably, none of the cited studies have comprehensively examined the influence of all eight muscle groups in the lower limbs on falls in older adults. Additionally, most of the available studies did not evaluate muscle strength and power using the isokinetic dynamometer* [2,7,9,12] *(considered the gold standard for assessing muscle strength* [16]*), and they often focused on isolated muscle groups or a subset of the lower limb muscles assessment* [1,2,6–8,10,13]*, which may not provide a complete understanding of overall muscle function.*

*Therefore, understanding the simultaneous association between all eight muscle groups of the lower limbs and falls in older adults is crucial for developing an effective fall prevention program. Our study addresses this gap by investigating by isokinetic dynamometer the influence of ankle plantarflexors, ankle dorsiflexors, knee flexors, knee extensors, hip flexors, hip extensors, hip abductors and hip adductors muscle strength and muscle power on falls in older adults, aiming to determine which muscle groups are more predictive of fall risk in this population. This comprehensive approach may offer insights for developing targeted and effective strategies in fall prevention and rehabilitation. Based on the existing literature* [1–15]*, our hypothesis is that our findings will align with previous findings*[1,2,7–11,15]*, indicating that ankle muscles will be more predictive of the occurrence of falls in older adults.”*

**Comment#4**

**Methods, Line 102-106: Authors stated one of the inclusion criteria as "no marked cognitive impairment examined by the Mini-Mental State Examination (MMSE) (cut-off point of 20 points for illiterate individuals; 25 points for people with 1 to 4 years of schooling; 26.5 for 5 to 8 years of schooling; 28 for those with 9 to 11 years of schooling; and 29 for more than 11 years of schooling). Please provide justification for considering these cut-off points.**

**Answer:** Thank you for your question. We have adopted the cutoff points for cognitive impairment assessment established by the Mini-Mental State Examination (MMSE), based on the recent research conducted by Brucki et al., 2003 (https://doi.org/10.1590/S0004-282X2003000500014). This research was conducted in the Brazilian context, and the criteria employed are specifically tailored to the reality of our sample, comprising Brazilian older adults. We selected these criteria to guarantee a more precise assessment of cognitive impairment, considering variations in educational levels within our sample. This approach is designed to better align with cognitive patterns in the target population, ensuring the validity and representativeness of the study results.

**Comment#5**

**Discussion, please discuss the significant results obtained in Table 2.**

**Answer**: Thank you for the consideration. We discussed it on *page 17, lines 254-261*:

*“Comparisons between older adults who experienced a fall and those who did not revealed that non-fallers had significantly better isokinetic muscle strength in the hip abductors and adductors, as well as higher muscle power in hip abductors, hip flexors, and knee flexors compared to fallers. This finding is somewhat surprising, considering that several previous studies suggested that ankle muscle strength and power are more closely associated with falls in older adults* [1,2,7–11,15]*. On the other hand, Morcelli et al.* [6] *had already demonstrated that older fallers had significantly lower strength in hip extension, abduction, and adduction, as well as lower power in hip flexion, extension, and abduction compared to non-fallers,* *which is consistent with our findings.”*

**Comment#6**

**Discussion, the authors should provide the reason/reasons for the lack of significant relationships between strength and power of muscle groups in knee and ankle joints and falls in older adults.**

**Answer:** Thank you for the consideration. We discussed it on *page 18, lines 286-296*:

*“Contrasting with findings from prior studies* [1,2,7–15]*, our observations highlight a notable absence of significant associations between the strength and power of muscle groups in the knee and ankle joints and occurrences of falls in older adults. This discrepancy can be attributed to the fact that previous research did not include a comprehensive analysis of all eight muscle groups in the lower limbs and did not utilize the isokinetic dynamometer, acknowledged as the gold standard for assessing muscle strength* [16]*. These methodological limitations likely played a role in the disparities among study outcomes, posing a challenge in establishing coherent relationships between distinct muscle groups and the incidence of falls. Additionally, most research has focused exclusively on assessing the musculature of the ankle and/or knee, disregarding the analysis of hip muscles. This may explain why the importance of these muscles in functional performance and fall prevention in older adults has not been fully elucidated.”*

**Reviewer #2**

**Reviewer #2: The paper was on “Influence of lower limb isokinetic muscle strength and power on the occurrence of falls”. The article is generally well written and I came to the opinion that this article needs minor revision. I am including my comments and I will like the authors to address the following comments:**

**Answer:** We sincerely appreciate the time and effort invested by the reviewer in critically assessing our manuscript. The insightful feedback and constructive comments have significantly contributed to the improvement of our work.

**Abstract**

**• The manuscript does need a structured abstract with distinct headings.**

**Answer:** Thank you for your feedback. We have introduced the distinct headings in the abstract.

**• Some more information regarding participants' characteristics.**

**Answer:** Thank you for the observation. We have introduced more information regarding participants' characteristics. *Page 2, Lines 39-41*:

***“Results****: Participants, with a median age of 69 years (range 64-74), included 67% women, and 63.8% reported a sedentary lifestyle. Among them, 45,7% of older adults were classified as fallers.”*

**• More details regarding study results needed.**

**Answer:** Thank you for the observation. We have introduced more details regarding study results. *Page 2, Lines 39-44*:

***“Results****: Participants, with a median age of 69 years (range 64-74), included 67% women, and 63.8% reported a sedentary lifestyle. Among them, 45,7% of older adults were classified as fallers. Comparative analyses revealed that non-fallers displayed significantly superior isokinetic muscle strength in the hip abductors and adductors, along with higher muscle power in the hip abductors, hip flexors, and knee flexors compared to fallers.”*

**Introduction**

**• Page 3, lines 63-65: " …strength decreases by 1.8 to 2.0% per year and power by 3.2 to 3.7% per year. According to Frontera et al., there is a 20 to 30% reduction in the isokinetic strength of knee flexors and extensors at low and high speeds in twelve years". It would have been good the ages that the reductions occur in mentioned studies was highlighted more clearly.**

**Answer:** Thank you for the feedback. We have introduced this information on *Page 3, Lines 65-67*:

*“Muscle strength decreases by 1.8 to 2.0% per year and power by 3.2 to 3.7% per year in older adults (age range 65-89 years)* [17]*. According to Frontera et al.* [18]*, in sedentary men with initial mean age 65.4 years-old (age range 60-72 years), there was a 20 to 30% reduction in the isokinetic strength of knee flexors and extensors at low and high speeds in twelve years.”*

**• Page 4, lines 88-93: Objectives and hypotheses of the study must be stated clearly. Please revise.**

**Answer:** Thank you for your feedback. After carefully examining the literature, we added our hypothesisas follows:

*Page 4, lines 100 to 102: “**Based on the existing literature*[1–15]*, our hypothesis is that our findings will align with previous findings*[1,2,7–11,15]*, indicating that ankle muscles will be more predictive of the occurrence of falls in older adults.”*

**Methods**

**• Page 6, line 123: Some details of functional performance tests (sit to stand and walking speed) required.**

**Answer:** Thank you for the feedback. We have introduced this information on *Page 6, Lines 137-144*:

***“****To assess walking speed, a 4-meter distance was marked, and participants were instructed to walk at their usual pace. The time to complete the test was recorded in two attempts, with the shorter time to complete the walking assessment considered for scoring. The usual walking speed (m/s) was then calculated by dividing the distance covered in the test (4 meters) by the shortest time recorded for that course in seconds* [19–21]*. In the five-time sit-to-stand test, participants were asked to rise from a chair, starting from a seated position, with arms crossed over the chest, repeating the movement five times as quickly as possible. The elapsed time in seconds was recorded as a performance measure* [19–21]*.”*

**• Page 7, line 143: Pictures are useful to illustrate muscle strength and power assessment by Biodex system. Can this be included?**

**Answer:** Thank you very much for the observations. We have inserted a figure to illustrate muscle strength and power assessment by the Biodex system on *“Materials and Methods” section, Page 9, lines 188-191.*

**Discussion**

**• Based on the results of study “the fallers showed worse functional performance than non-fallers in the sit-to-stand and walking speed tests” I would suggest that this should be discussed as one of the findings of study in section for discussion. Can this be included?**

**Answer:** Thank you for the feedback. We have discussed these results on *Page 17, Lines 240-253*:

“*Our findings indicated that fallers exhibited worse functional performance than non-fallers in the sit-to-stand and walking speed tests. These findings align with various prior studies [22–27] that emphasized the significant association between slower walking speed and occurrences of falls in older adults. These results can be explained by the impact of aging on the gait of older individuals [28], characterized by changes in three-dimensional kinetics and kinematics, including reduced movement of the pelvis in the frontal and transverse planes, prolonged hip adduction, and increased knee extension peak during the support phase (hyperextension). These alterations, coupled with reduced ankle plantar flexion during the terminal support phase and decreased knee range of motion compared to younger individuals, contribute to the decrease in walking speed [28]. Additionally, previous studies have also highlighted the association between poorer performance in the sit-to-stand test and a higher risk of falls in older adults [29–31]. This relationship can be attributed to the influence of aging on this function [17,32], as evidenced by the greater difficulty that older individuals face when rising from a chair compared to younger counterparts [32,33]*.”

**REFERENCES:**

[1]	Whipple RH, Wolfson LI, Amerman PM. The relationship of knee and ankle weakness to falls in nursing home residents: An isokinetic study. J Am Geriatr Soc 1987;35:13–20.

[2]	Studenski S, Duncan PW, Chandler J. Postural Responses and Effector Factors in Persons with Unexplained Falls: Results and Methodologic Issues. J Am Geriatr Soc 1991;39:229–34. https://doi.org/10.1111/j.1532-5415.1991.tb01642.x.

[3]	Clynes MA, Edwards MH, Buehring B, Dennison EM, Binkley N, Cooper C. Definitions of Sarcopenia: Associations with Previous Falls and Fracture in a Population Sample. Calcif Tissue Int 2015;97:445–52. https://doi.org/10.1007/s00223-015-0044-z.

[4]	Scott D, Johansson J, McMillan LB, Ebeling PR, Nordstrom P, Nordstrom A. Associations of Sarcopenia and Its Components with Bone Structure and Incident Falls in Swedish Older Adults. Calcif Tissue Int 2019. https://doi.org/10.1007/s00223-019-00540-1.

[5]	Moreland JD, Richardson JA, Goldsmith CH, Clase CM. Muscle weakness and falls in older adults: a systematic review and meta-analysis. J Am Geriatr Soc 2004;52:1121–9. https://doi.org/10.1111/j.1532-5415.2004.52310.x.

[6]	Morcelli MH, Crozara LF, Rossi DM, LaRoche DP, Ribeiro Marques N, Hallal CZ, et al. Hip muscles strength and activation in older fallers and non-fallers. Isokinet Exerc Sci 2014;22:191–6. https://doi.org/10.3233/IES-140538.

[7]	Lord SR, Clark RD, Webster IW. Physiological factors associated with falls in an elderly population. J Am Geriatr Soc 1991;39:1194–200.

[8]	Wolfson L, Judge J, Whipple R, King M. Strength is a major factor in balance, gait, and the occurrence of falls. J Gerontol A Biol Sci Med Sci 1995;50 Spec No:64–7.

[9]	Daubney ME, Culham EG. Lower-extremity muscle force and balance performance in adults aged 65 years and older. Phys Ther 1999;79:1177–85. https://doi.org/10.1016/0197-4580(89)90010-9.

[10]	Skelton DA, Kennedy J, Rutherford OM. Explosive power and asymmetry in leg muscle function in frequent fallers and non-fallers aged over 65. Age Ageing 2002;31:119–25.

[11]	Pinho L, Dias RC, Freire MTF, Tavares CF, Dias JMD. Isokinetic evaluation of hip and ankle muscle function among elderly people who suffer falls. Brazilian J Phys Ther 2005;9:93–9.

[12]	MacRae PG, Lacourse M, Moldavon R. Physical Performance Measures That Predict Faller Status in Community-Dwelling Older Adults. J Orthop Sport Phys Ther 1992;16:123–8. https://doi.org/10.2519/jospt.1992.16.3.123.

[13]	Crozara LF. Análise comparativa de variáveis biomecânicas entre as mulheres idosas caidoras e não caidoras. Universidade Estadual Paulista, 2012.

[14]	Pavol MJ, Owings TM, Foley KT, Grabiner MD. Influence of lower extremity strength of healthy older adults on the outcome of an induced trip. J Am Geriatr Soc 2002;50:256–62.

[15]	Epro G, McCrum C, Mierau A, Leyendecker M, Brüggemann G-P, Karamanidis K. Effects of triceps surae muscle strength and tendon stiffness on the reactive dynamic stability and adaptability of older female adults during perturbed walking. J Appl Physiol 2018;124:1541–9. https://doi.org/10.1152/japplphysiol.00545.2017.

[16]	McCarthy EK, Horvat MA, Holtsberg PA, Wisenbaker JM. Repeated Chair Stands as a Measure of Lower Limb Strength in Sexagenarian Women. Journals Gerontol Ser A Biol Sci Med Sci 2004;59:1207–12. https://doi.org/10.1093/gerona/59.11.1207.

[17]	Skelton DA, Greig CA, Davies JM, Young A. Strength Power and Related Functional Ability of Healthy People Aged 65-89 Yrs - Skelton 1994.pdf 1994:371–7.

[18]	Frontera W, Hughes VA, Fielding RA, Fiatarone M, Evans W, Roubenoff R. Aging of Skeletal Muscle: A 12-yr longitudinal study. J Appl Physiol J Gerontol A Biol Sci Med Sci J Appl Physiol 2000;88:1321–6.

[19]	Guralnik JM, Simonsick EM, Ferrucci L, Glynn RJ, Berkman LF, Blazer DG, et al. A short physical performance battery assessing lower extremity function: association with self-reported disability and prediction of mortality and nursing home admission. J Gerontol Med Sci 1994;49:M85-94. https://doi.org/10.1093/geronj/49.2.M85.

[20]	Nakano MM, Diogo MJDe, Filho WJ. Summary for Policymakers. Clim. Chang. 2013 – Phys. Sci. Basis, Cambridge University Press; 2014, p. 1–30. https://doi.org/10.1017/CBO9781107415324.004.

[21]	Guralnik JM, Ferrucci L, Simonsick EM, Salive ME, Wallace RB. Lower-extremity function in persons over the age of 70 years as a predictor of subsequent disability. N Engl J Med 1995;332:556–61. https://doi.org/10.1056/NEJM199503023320902.

[22]	Quach L, Galica AM, Jones RN, Procter-Gray E, Manor B, Hannan MT, et al. The nonlinear relationship between gait speed and falls: the Maintenance of Balance, Independent Living, Intellect, and Zest in the Elderly of Boston Study. J Am Geriatr Soc 2011;59:1069–73. https://doi.org/10.1111/j.1532-5415.2011.03408.x.

[23]	Viccaro LJ, Perera S, Studenski SA. Is timed up and go better than gait speed in predicting health, function, and falls in older adults? J Am Geriatr Soc 2011;59:887–92. https://doi.org/10.1111/j.1532-5415.2011.03336.x.

[24]	Morita M, Takamura N, Kusano Y, Abe Y, Moji K, Takemoto T-I, et al. Relationship between falls and physical performance measures among community-dwelling elderly women in Japan. Aging Clin Exp Res 2005;17:211–6.

[25]	Montero-Odasso M, Schapira M, Soriano ER, Varela M, Kaplan R, Camera LA, et al. Gait velocity as a single predictor of adverse events in healthy seniors aged 75 years and older. J Gerontol A Biol Sci Med Sci 2005;60:1304–9. https://doi.org/10.1093/gerona/60.10.1304.

[26]	Tiedemann A, Shimada H, Sherrington C, Murray S, Lord S. The comparative ability of eight functional mobility tests for predicting falls in community-dwelling older people. Age Ageing 2008;37:430–5. https://doi.org/10.1093/ageing/afn100.

[27]	Ward RE, Leveille SG, Beauchamp MK, Travison T, Alexander N, Jette AM, et al. Functional performance as a predictor of injurious falls in older adults. J Am Geriatr Soc 2015;63:315–20.

[28]	Judge JO, Davis RB, Ounpuu S. Step length reductions in advanced age: the role of ankle and hip kinetics. J Gerontol A Biol Sci Med Sci 1996;51:M303–12. https://doi.org/10.1093/gerona/51A.6.M303.

[29]	Guzman RA, Porcel Melian H, Cordier B, Adul Silvestre R. [Differences in biomechanical parameters during sit to stand transfer between elderly with and without history of frequent falls]. Rev Esp Geriatr Gerontol 2010;45:267–73. https://doi.org/10.1016/j.regg.2010.05.002.

[30]	Chorin F, Cornu C, Beaune B, Frere J, Rahmani A. Sit to stand in elderly fallers vs non-fallers: new insights from force platform and electromyography data. Aging Clin Exp Res 2016;28:871–9. https://doi.org/10.1007/s40520-015-0486-1.

[31]	Buatois S, Miljkovic D, Manckoundia P, Gueguen R, Miget P, Vancon G, et al. Five times sit to stand test is a predictor of recurrent falls in healthy community-living subjects aged 65 and older. J Am Geriatr Soc 2008;56:1575–7. https://doi.org/10.1111/j.1532-5415.2008.01777.x.

[32]	Landers KA, Hunter GR, Wetzstein CJ, Bamman MM. The interrelationship among muscle mass , strength , and the ability to perform ... 2001;56:443–8.

[33]	Landi F, Calvani R, Tosato M, Martone AM, Fusco D, Sisto A, et al. Age-Related Variations of Muscle Mass, Strength, and Physical Performance in Community-Dwellers: Results From the Milan EXPO Survey. J Am Med Dir Assoc 2017;18:88.e17-88.e24. https://doi.org/10.1016/j.jamda.2016.10.007.
